# Collection on total-body PET

**DOI:** 10.1186/s40658-023-00550-x

**Published:** 2023-07-10

**Authors:** Kuangyu Shi, Charalampos Tsoumpas

**Affiliations:** 1 Department of Nuclear Medicine, Bern University Hospital, University of Bern, Bern, Switzerland; 2 Department of Nuclear Medicine and Molecular Imaging, University of Groningen, University Medical Center Groningen, Groningen, The Netherlands

With the prompt appearance of extended axial field of view positron emission tomographs (LAFOV PET), an extensive range of prospects become possible in molecular diagnostics. An advantage using LAFOV PET is the increase of sensitivity, which can be more than 10 times higher for 1-m-long PET [1] and 40 times for a 2-m-long PET scanner [2], yet it is essential to state that these figures depend on the patient, organs of study and tracer distribution.

On the one hand, the axial extension of the scanners and their increased sensitivity offer a mixture of benefits:

- Ultra-fast scanning [3]
- Simultaneous imaging of more organs [4]
- Scanning kinetics with wider temporal range (e.g. delayed imaging or very short frames) [5]
- Enhanced image quality [6]
- Scanning with appreciably lower injected radioactivity [7]
- Enhanced support of systems biology/medicine [8]
- Enhanced support of dosimetry and theranostics [9]

On the other hand, LAFOV PET scanners also brought new methodological challenges [10]. The tremendous numbers of detectors strongly increased the complexity and data volume. It becomes more challenging to deal with large heterogeneity inside the scanner, for example, due to the oblique lines of response.

Several LAFOV PET scanners across the world have been already installed and been in use creating a wide range of exciting research [11].

EJNMMI also offers a collection on total body PET [11] which is available at https://link.springer.com/journal/259/collections.

We look forward to receiving submissions of work by researchers from all around the world.
